# Case Report: Denys–Drash Syndrome With *WT1* Causative Variant Presenting as Atypical Hemolytic Uremic Syndrome

**DOI:** 10.3389/fped.2020.605889

**Published:** 2020-12-18

**Authors:** Cheng Cheng, Lizhi Chen, Sijia Wen, Zhilang Lin, Xiaoyun Jiang

**Affiliations:** Department of Pediatrics, The First Affiliated Hospital of Sun Yat-sen University, Guangzhou, China

**Keywords:** denys-drash syndrome (DDS), pseudohermaphroditism, atypical hemolytic uremic syndrome (aHUS), case report [publication type], wilms tumor 1 gene (WT1)

## Abstract

The *WT1* variant is confirmed to be pathogenic for Denys–Drash syndrome (DDS), a rare disorder characterized by early-onset nephrotic syndrome and renal failure, pseudo-hermaphroditism, and a high risk of Wilms' tumor. Several cases of DDS presenting with atypical hemolytic uremic syndrome (aHUS) have been reported. Here we report the case of a 2-year-old child who was diagnosed with *WT1* missense variant, associated with DDS and initial presentation of aHUS. Complement factor H autoantibodies were negative. Complement regulatory system-related gene variants were not found, but a *de novo* heterozygous c.754G>A missense variant in exon 9 of *WT1* gene was detected, resulting in a p. Asp252Asn substitution, by next-generation sequencing. The patient was a female morphologically but proved to be a genetic male because of karyotype 46, XY with normally developed female external genitalia. Bilateral nephrectomy and renal transplantation were performed 1 year later, and there was no recurrence of aHUS at 10 months after transplantation.

## Introduction

Denys–Drash syndrome (DDS) is a rare genetic disorder characterized by early-onset nephrotic syndrome that rapidly progresses to renal failure during the first few years of life, pseudo-hermaphroditism, and a high risk of developing Wilm's tumor (1). Wilms' tumor 1 (*WT1*) gene variants have been shown to be pathogenic for DDS. The *WT1* gene encodes proteins that regulate progenitor cells and their differentiation, especially those of the gonads, uteri, and kidneys (2, 3). The initial manifestations of DDS can be vague and indistinguishable. Here we report the case of a 2-year-old girl who initially presented with atypical hemolytic uremic syndrome (aHUS) and was eventually diagnosed with DDS with *WT1* gene heterozygous variant. HUS is a triad of microangiopathic hemolytic anemia, thrombocytopenia, and AKI. aHUS is known to be caused by genetic alterations in the complement alternative pathway or formation of complement factor H (CFH) autoantibodies, leading to complement system dysregulation (4, 5).

## Case Description

**Figure 1:**
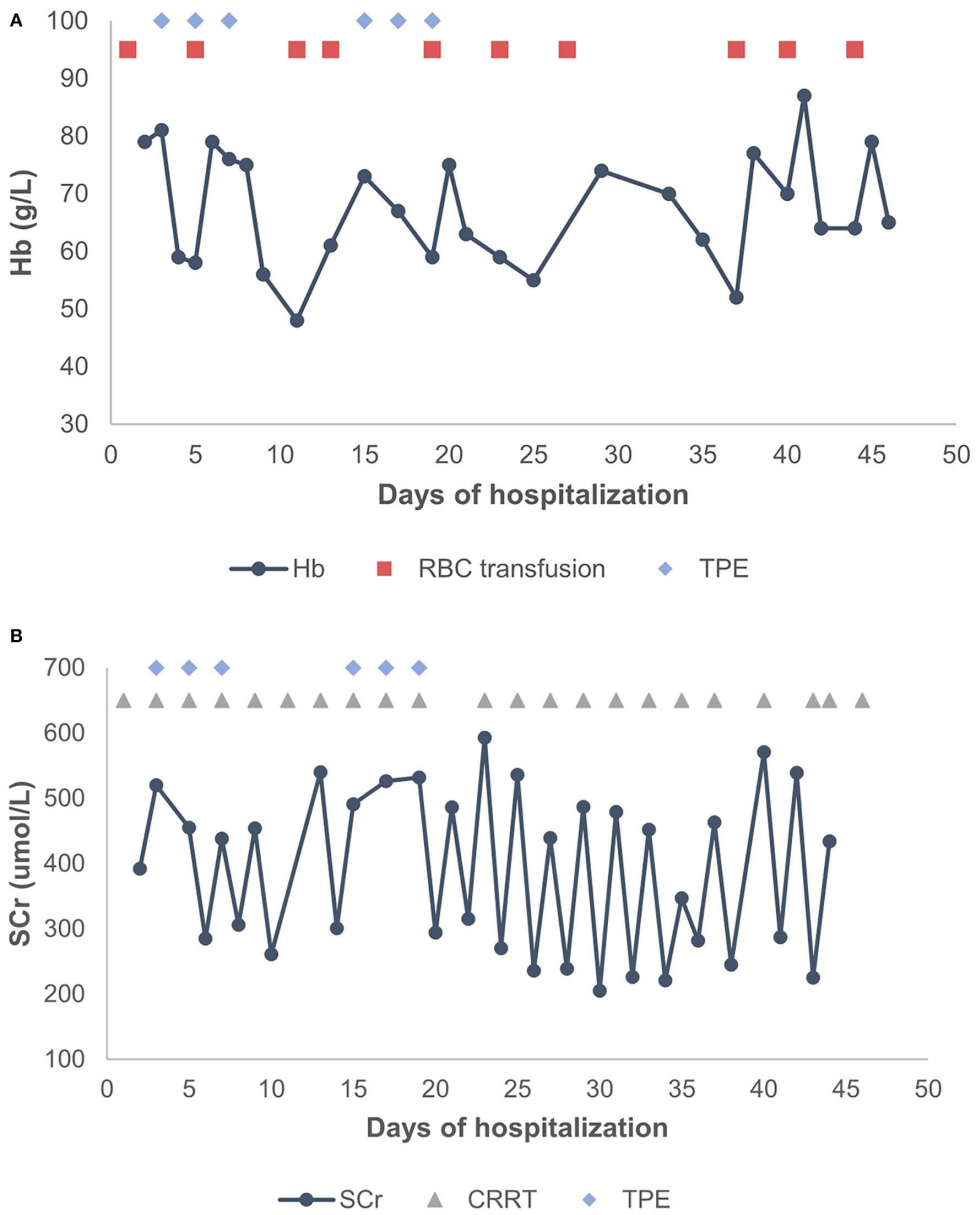
**(A)** Changes in Hb levels after treatment. **(B)** Changes in SCr levels after treatment. TPE, therapeutic plasma exchange; CRRT, continuous renal replacement therapy; RBC, red blood cell; Hb, hemoglobin; SCr, serum creatinine.

**Figure 2:**
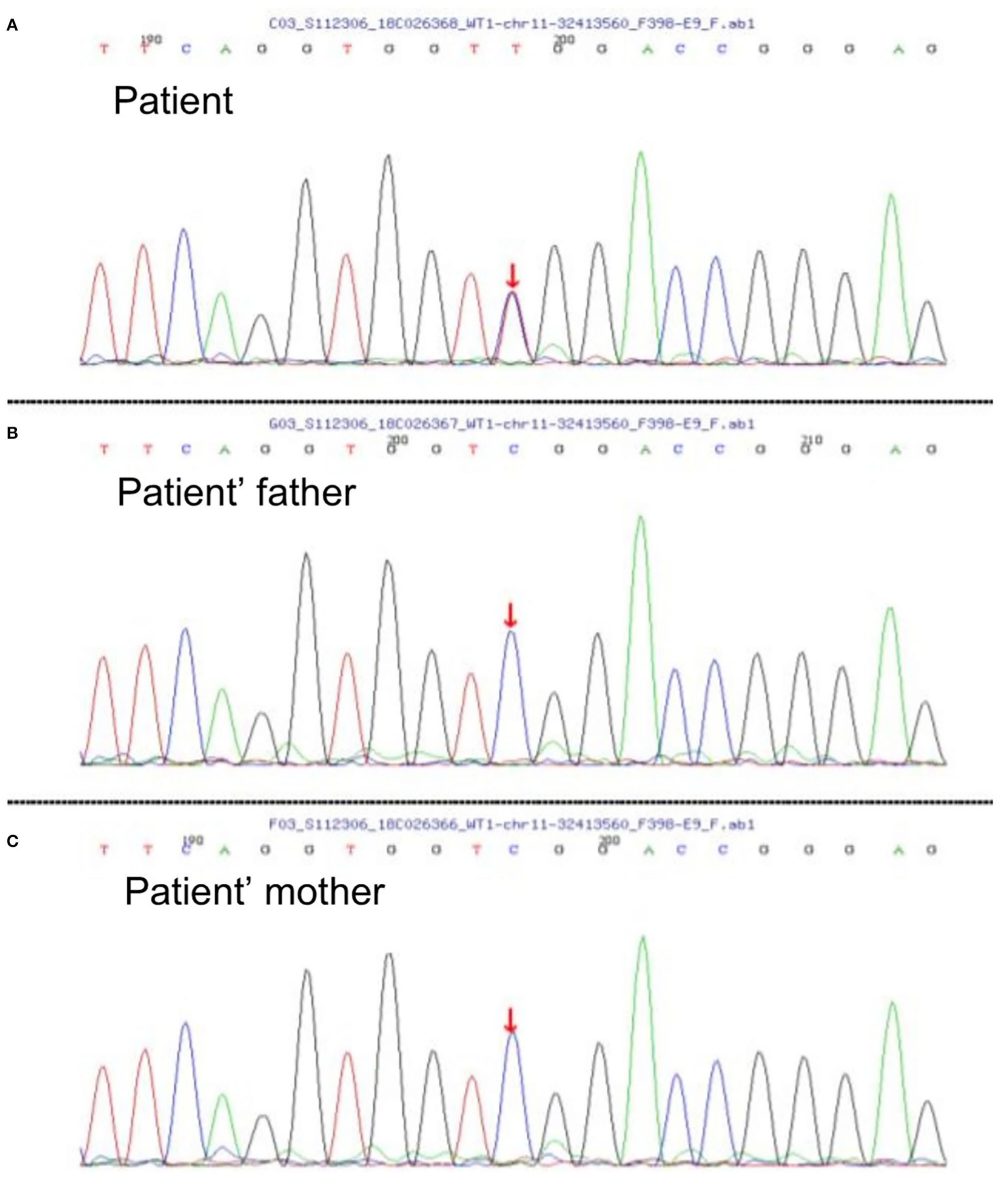
**(A)** Diagram of the patient's *de novo* heterozygous c.754G>A missense variant in exon 9 of *WT1* gene; nucleotide 754 in the coding region was altered from guanine to adenine, resulting in a change of amino acid 252 from aspartic to asparagine. This missense variant has been reported to be pathogenic to Denys–Drash syndrome (DDS) according to the Human Gene Mutation Database, and it was evaluated to be pathogenic to DDS according to the American College of Medical Genetics and Genomics standards and guidelines. **(B,C)** Diagrams of *WT1* genes of the patient's parents showing no variation. Since reverse reads were used by Sanger sequencing, the bases shown in the peak diagram are the reverse complementary sequence C>T of the altered bases.

A 2-year-old girl had diffusely distributed rashes followed by abdominal pain, non-bloody loose stools (once a day, which resolved in 2 days), anasarca, and anuria for a month. The patient was admitted to a local hospital on October 18, 2018 with anemia (hemoglobin, 87 g/L), thrombocytopenia (platelet, PLT 82 × 10^9^/L), stage 3 acute kidney injury (AKI) according to the guidelines of Kidney Disease: Improving Global Outcomes (KDIGO) (serum creatinine 961 μmol/L; creatinine clearance, 4.6 ml/min/1.73m^2^; and blood urea nitrogen 77.2 mmol/L) (6). Initial urinalysis showed urine protein 3+; 24-h proteinuria was 0.16 g (24-h urine amount: 40 ml), and other blood biochemistry analyses demonstrated hypoalbuminemia (albumin, ALB 30.5 g/L) and hyperlipidemia (total cholesterol, TC 6.42 mmol/L). There was no abdominal pain, loose stools, or vomiting. Continuous renal replacement therapy (CRRT) was initiated the first time. The child was transferred to our hospital 7 days later. Physical examination revealed hypertension (blood pressure was initially normal), pallor, generalized edema, and hepatomegaly. Routine blood tests showed moderate anemia with increased reticulocyte percentage (5.39%), while PLT returned to baseline levels. Urinalysis showed 1+ hematuria and 3+ proteinuria but improved hypoalbuminemia (ALB 31 g/L) and hyperlipidemia (TC, 5.8 mmol/L). Other blood chemistry analyses were consistent with KDIGO stage 3 AKI, and lactate dehydrogenase was elevated (386 U/L). Helmet erythrocytes were observed in peripheral blood smears. Coombs test and paroxysmal nocturnal hemoglobinuria screening tests were negative. Platelet activation tests and complement C3 levels were normal. Stool culture was negative for *Escherichia coli*. aHUS was, thus, diagnosed. Therefore, the patient underwent alternate-day CRRT and six sequences of therapeutic plasma exchange. Hemolysis still progressed, and renal function did not recover after treatment (Figure 1). Eculizumab was inaccessible since it had just been approved in the Chinese market, and renal biopsy was not performed due to coagulation issues. However, further measures were taken to determine the cause of aHUS. CFH autoantibodies were negative. Next-generation sequencing assay for the nephrology panel (MyGenostics) was performed using peripheral blood samples from the patient and her parents. Genes that are known to be associated with aHUS, including complement regulatory protein-coding genes and other related genes (*C3, C4, C5, CFB, CFH, CFI CFHR1, CFHR3, CFHR4, CFHR5, THBD, PLG*, and *DGKE*), were included. However, no variants were detected. Unexpectedly, a *de novo* heterozygous c.754G>A missense variant in exon 9 of the *WT1* gene (NM_001198551.1: c.754G>A) was detected, resulting in a p. Asp252Asn substitution (NP_001185480.1: p. Asp252Asn), and this was further verified by Sanger sequencing (Figure 2). Variant analysis according to the American College of Medical Genetics and Genomics standards and guidelines revealed PS1 (sequence variation had previously been reported to be pathogenic for DDS in the Human Gene Mutation Database) + PS2 (*de novo* and no family history) + PM2 (sequence variation absent from controls), which was regarded as pathogenic for DDS (RefSNP number: rs28941778). The karyotype of this patient was suspected to be 46, XY. As described earlier, DDS is a condition characterized by the triad of nephropathy, genitalia abnormality, and a high risk of Wilm's tumor (1). The patient was a female but later proved to be a genetic male owing to the 46, XY karyotype; however, normally developed female external genitalia was observed. Abdominal computed tomography did not reveal cryptorchidism or abnormal renal mass. There was no improvement in renal function following therapy; hence, the patient received regular peritoneal dialysis and underwent renal transplantation 1 year later; however, prior to that, bilateral nephrectomy was performed for the prevention of Wilm's tumor. Gross specimen examination demonstrated many dysplastic glomeruli and partial sclerotic glomeruli, protein casts, and calcium depositions in the renal tubes, with vacuolar degeneration in the epithelium of the proximal tubes, thickened arteriolar walls and narrowed lumens, and interstitial inflammation infiltration. There was no recurrence of aHUS 10 months after transplantation.

## Discussion

DDS is a rare genetic disorder characterized by early-onset nephrotic syndrome that rapidly progresses to renal failure during the first few years of life, pseudo-hermaphroditism, and a high risk of developing Wilm's tumor (1). It has been proven that constitutional variants in the zinc finger (ZF) motif of the *WT1* gene are associated with DDS (2). The *WT1* gene is located at chromosome 11p13 and contains 10 coding exons. It encodes a transcription factor that contains four Cys2-His2 ZF DNA-binding domains at the C-terminus and a proline/glutamine-rich regulatory domain at the N-terminus (7). There are two alternative splicing resulting in four distinct *WT1* isoforms: 17 amino acids encoded by exon 5 are inserted into the proline-terminus, producing *WT1* +17AA/-17AA isoforms, and three amino acids lysine-threonine-serine (KTS) encoded by exon 9 are inserted between ZF3 and ZF4, producing *WT1* +KTS/-KTS isoforms (8). The *WT1* gene is essential for the development of both kidneys and the genital system, and it is expressed in renal podocytes throughout life (9). Most of the *WT1* variations in DDS are heterozygous germ-line variations that occur in exon 8 or 9, encoding protein ZF2 or ZF3, with a frequent variant of p. Arg394Trp substitution, leading to either DNA binding capacity alteration or isoform imbalance (2, 10).

The case of DDS that we report here had a *de novo* heterozygous c.754G>A; p. Asp252Asn missense variant in exon 9 of *WT1*. Unexpectedly, the patient initially presented with symptoms of aHUS until the diagnosis of DDS was made based on early-onset nephropathy, male pseudo-hermaphroditism, and *WT1* sequence variation. Enterocolitis caused by Shiga toxin-producing *Escherichia coli* accounts for most of the HUS cases in children (3). In contrast, the atypical form of HUS is often caused by genetic alterations in the complement alternative pathway or by CFH autoantibody formation, leading to complement system overactivation (4). Non-Shiga toxin-induced diarrhea prior to aHUS has been reported in some pediatric cases (11, 12). Our case did not have any underlying diseases associated with HUS. However, she neither harbored a variant related to the complement regulatory system nor synthesized CFH antibody.

This is the first case of DDS presenting with HUS reported in China; four similar cases have been reported previously in other countries (Table 1). Manivel et al. first noted a patient with a 46, XY karyotype who was born with ambiguous genitalia and had developed HUS at 26 months of age (13). Renal biopsy was consistent with the diagnosis of HUS, while bilateral nephrectomy demonstrated diffuse mesangial sclerosis (DMS) at 32 months of age. A few decades later, Sherbotie et al. described two patients with DDS who had also developed features of aHUS (14). The first case was a 13-month-old boy who developed aHUS 1 month after nephrotic syndrome and urinary tract infection with *Citrobacter* and *Enterococcus*. The other patient was a 16-month-old boy who manifested aHUS and DDS at the same time. Normal serum C3 levels were revealed, and *WT1* exon 9 missense variants were identified in both 46, XY males with undescended testes and grade III hypospadias. Renal failure was irreversible, and transplantations were eventually carried out in both cases. The renal pathologies revealed diffuse sclerosis. Alge et al. recently reported an 8-month-old female infant who had aHUS as the initial manifestation of DDS (15). Whole exome sequencing identified a pathogenic *de novo* heterozygous variant in the *WT1* gene with predisposing variants in the *CFH* gene and an unknown variant in the *DGKE* gene. Acquired causes of aHUS were ruled out. Karyotyping confirmed that the patient was 46, XX with normal external genitalia, but without ovaries. In contrast to the previously reported cases, our patient was a 46, XY child with female physical appearance and external genitalia. In addition, our case had normal C3 levels, as in the case reported by Sherbotie et al. which is not uncommon in aHUS (11). *WT1* sequence variants associated with DDS were detected in our patient as well as in the cases reported by Sherbotie et al. and Alge et al. Causative variants related to aHUS and autoantibodies against CFH were screened in our case and the case of Alge et al. but no direct pathogenic result was obtained.

**Table 1:** Clinical finding in cases of *WT1* variants associated with Denys–Drash syndrome (DDS) presenting as HUS.

	**Present study**	**Patient 1**	**Patient 2**	**Patient 3**	**Patient 4**
Study	–	Manivel et al. (13)	Sherbotie et al. (14)	Sherbotie et al. (14)	Alge et al. (15)
Age at onset	2 years	26 months	13 months	16 months	8 months
Karyotype	46, XY	46, XY	46, XY	46, XY	46, XX
Morphological sex	Female	–	Male	Male	Female
Initial manifestation	Rashes, abdominal pain, non-bloody loose stools, edema, anuria	HUS	UTI, edema	Edema, vomiting, decreased urine output	Oliguria, hypertension, vomiting
Genital abnormality	Female external genitalia	Ambiguous external genitalia, bilateral Mullerian and right Wolffian	Grade III hypospadias with undescended testes	Grade III hypospadias with bifid scrotum, undescended testes	Absence of ovary
Nephrotic syndrome	No	–	A month before HUS	At presentation with HUS	No
DDS diagnosed before HUS	No	No	No	No	No
C3 level	Normal	–	Normal	Normal	Decreased
Renal pathology	Gross specimens: DMS and glomerular dysplasia	Initial biopsy: MPGN Gross specimens: DMS and metanephric hamartoma	DMS without microangiopathic glomerulopathy	DMS without microangiopathic glomerulopathy	Initial biopsy: 3/8 glomeruli with globally sclerotic and the rest with active TMA Gross specimens: DMS
Wilm's tumor	No	No	No	No	No
Treatment	Plasma exchange	–	Plasmapheresis	–	Plasma infusions/exchange, eculizumab
Renal function	Irreversible	Irreversible	Irreversible	Irreversible	Irreversible
Outcome	Bilateral nephrectomy and renal transplantation at 3 years old, 10 months no recurrence	Bilateral nephrectomy, gonadectomy, and renal transplantation at 32 months old	PD; bilateral nephrectomy and renal transplantation at 2.5 years old, 4.5 years no recurrence	PD and HD; renal transplantation at 5 years old, 18 months no recurrence	PD; bilateral nephrectomy at 13 months old
Complement abnormality	Not found	–	–	–	Autoantibody (-) *CFH* H3 haplotype with three SNPs^a^
*WT1* variation	Heterozygous, p. Asp252Asn in exon 9	–	Heterozygous, IVS9+111 C>T	Heterozygous, p. Arg394Trp in exon 9	Heterozygous, p. Arg394Trp in exon 9
Clinvar interpretation	Pathogenic	–	Variant not in clinvar	Pathogenic	Pathogenic

*HUS, hemolytic uremic syndrome; DMS, diffuse mesangial sclerosis; UTI, urinary tract infection; SNPs, single-nucleotide polymorphisms; MPGN, membranoproliferative glomerulonephritis; PD, peritoneal dialysis; HD, hemodialysis*.

a *c.1-332 C>T in the promoter, silent mutation at p. Gln672, and p. Glu936Asp substitution*.

Among the existing cases, two underwent renal biopsies at the time of HUS diagnosis, and thrombotic microangiopathy (TMA) was biopsy-proven. At 5 to 6 months later, DMS was demonstrated by bilateral nephrectomy when transplantation was performed, suggesting that HUS occurred prior to DDS. Additionally, DMS was also evident in two other cases a few years after disease onset. Unfortunately, our case was diagnosed based on clinical manifestations and genetic sequencing since the patient was unable to tolerate renal biopsy at the beginning. Gross pathology after nephrectomy revealed glomerular dysplasia and sclerosis.

*WT1*-encoded proteins are crucial in regulating cell growth and maintaining the normal function of renal podocytes, the cells that comprise the outer layer of the glomerular filtration barrier. Sequence variants that influence ZFs and disturb the splicing of +KTS/-KTS isoforms result in dedifferentiation, abnormal proliferation, or morphological alteration in mutant podocytes (16, 17). Although the mechanisms are still not well-understood, studies have proposed that reduced *WT1* expression, platelet-derived growth factor-α (PDGF-α), and transforming growth factor-β1 (TGF-β1) overexpression and upregulated *Pax-2* expression play roles in this regard (17). Consequently, abnormal podocytes disrupt the integrity of the glomerular filtration barrier, leading to remarkable proteinuria in DDS, while nephrotic-range proteinuria promotes the release of various procoagulants, creating an environment with a high risk of TMA (18). A recent study also found that podocyte injuries are commonly seen in renal TMA (19). Vascular endothelial growth factor (VEGF) secreted by podocytes is important for the survival of endothelial cells, podocytes, and mesangial cells. Its concentration is maintained by the appropriate size selectivity of the podocyte slit diaphragm; however, this selectivity may be affected by severe proteinuria (20). A study in adult mice has shown that a reduction in the podocyte-derived VEGF level is sufficient to induce profound TMA (21), while children with different nephropathies accompanied by nephrotic-range proteinuria have been reported to have aHUS as a complication (22). In addition, podocyte-derived VEGF participates in complement activity regulation, whereas dysregulation of complement pathways predisposes to TMA (23). Thus, emerging findings suggest that decreased VEGF concentration due to podocyte dysfunction contributes to the development of renal TMA (21, 22). Among the five above-mentioned cases of DDS with aHUS, four harbored causative variants in exon 9 of the *WT1* gene. The two cases described by Sherbotie *et al*. were diagnosed with nephrotic syndrome, while our patient and the case reported by Alge et al. had urine protein 3+ and 4+ by dipstick analysis, respectively. We thus hypothesize that podocytopathy in *WT1* variants may be one of the pathogenic factors contributing to the occurrence of aHUS.

The limitations of this case report include the following: [1] renal pathology was not obtained at diagnosis, but it is of importance in understanding disease progression and distinguishing the initiating etiology and [2] multiplex ligation-dependent probe amplification analysis of *CFHR1/CFHR3* deletion and *CFH/CFHR1* hybrid gene, two important predisposing variants of aHUS that could not be detected by standard sequencing (24, 25), was not performed. The etiology and pathogenesis of DDS with *WT1* variation presenting as aHUS is still unknown. The case reported here adds value to existing cases as it provides evidence that the association between DDS and aHUS is probably not coincidental. Genetic sequencing is useful in determining the underlying disease or comorbidity when both genetic and acquired causes of aHUS are ruled out. Whether DDS is a predisposing factor for aHUS or whether these conditions comprise another clinical syndrome requires further study.

## Patient Perspective

The following patient perspective was provided by the patient's parents.

“It was very sudden that our child became ill and deteriorated unexpectedly quickly. It had been nearly a month since the disease started, but we still decided to take a chance for therapeutic plasma exchange, although renal function failed to recover. Later, the WES and karyotyping results were astonishing. We transitioned from hemodialysis to peritoneal dialysis for long-term renal replacement while we were preparing and waiting for transplantation. Renal transplantation was successful, and our child is currently receiving regular anti-rejection treatments. We hope that our case will add new findings and contribute to the learning of these rare diseases.”

## Data Availability Statement

The original contributions presented in the study are included in the article/Supplementary Materials, further inquiries can be directed to the corresponding author/s.

## Ethics Statement

Written informed consent was obtained from the minor(s)' legal guardian/next of kin for the publication of any potentially identifiable images or data included in this article.

## Patient Consent

Informed consent was obtained from the patient's parents for the publication of this case report.

## Author Contributions

XJ conceptualized, designed the study, reviewed, and revised the manuscript. LC, CC, SW, and ZL collected, analyzed, interpreted clinical, imaging, genetic data, and were responsible for manuscript writing. All the authors contributed to the manuscript and approved the final version.

## Conflict of Interest

The authors declare that the research was conducted in the absence of any commercial or financial relationships that could be construed as a potential conflict of interest.

## References

[1] Denys P Malvaux P Van Den Berghe H Tanghe W Proesmans W Association d'un syndrome anatomo-pathologique de pseudohermaphrodisme masculin, d'une tumeur de Wilms, d'une nephropathie parenchymateuse et d'un mosaicisme XX/XY [Association of an anatomo-pathological syndrome of male pseudohermaphroditism, Wilms' tumor, parenchymatous nephropathy and XX/XY mosaicism]. Arch Fr Pediatr. (1967) 24:729–39. 4292870

[2] Pelletier J Bruening W Kashtan CE Mauer SM Manivel JC Striegel JE. Germline mutations in the Wilms' tumor suppressor gene are associated with abnormal urogenital development in Denys-Drash syndrome. Cell. (1991) 67:437–47. 10.1016/0092-8674(91)90194-4 1655284

[3] Lipska BS Ranchin B Iatropoulos P Gellermann J Melk A Ozaltin F. Genotype-phenotype associations in WT1 glomerulopathy. Kidney Int. (2014) 85:1169–78. 10.1038/ki.2013.519 24402088

[4] Cody EM Dixon BP. Hemolytic uremic syndrome. Pediatr Clin North Am. (2019) 66:235–46. 10.1016/j.pcl.2018.09.011 30454746

[5] Dixon BP Gruppo RA. Atypical hemolytic uremic syndrome. Pediatr Clin North Am. (2018) 65:509–25. 10.1016/j.pcl.2018.02.003 29803280

[6] Barry R James MT. Guidelines for classification of acute kidney diseases and disorders. Nephron. (2015) 131:221–6. 10.1159/000441425 26554580

[7] Call KM Glaser T Ito CY Buckler AJ Pelletier J Haber DA. Isolation and characterization of a zinc finger polypeptide gene at the human chromosome 11 Wilms' tumor locus. Cell. (1990) 60:509–20. 10.1016/0092-8674(90)90601-A 2154335

[8] Haber DA Sohn RL Buckler AJ Pelletier J Call KM Housman DE. Alternative splicing and genomic structure of the Wilms tumor gene WT1. Proc Natl Acad Sci USA. (1991) 88:9618–22. 10.1073/pnas.88.21.9618 1658787 PMC52769

[9] Pritchard-Jones K Fleming S Davidson D Bickmore W Porteous D Gosden C. The candidate Wilms' tumour gene is involved in genitourinary development. Nature. (1990) 346:194–7. 10.1038/346194a0 2164159

[10] Little M Holmes G Bickmore W van Heyningen V Hastie N Wainwright B. DNA binding capacity of the WT1 protein is abolished by Denys-Drash syndrome WT1 point mutations. Hum Mol Genet. (1995) 4:351–8. 10.1093/hmg/4.3.351 7795587

[11] Noris M Caprioli J Bresin E Mossali C Pianetti G Gamba S. Relative role of genetic complement abnormalities in sporadic and familial aHUS and their impact on clinical phenotype. Clin J Am Soc Nephrol. (2010) 5:1844–59. 10.2215/CJN.02210310 20595690 PMC2974386

[12] Sellier-Leclerc AL Fremeaux-Bacchi V Dragon-Durey MA Macher MA Niaudet P Guest G. Differential impact of complement mutations on clinical characteristics in atypical hemolytic uremic syndrome. J Am Soc Nephrol. (2007) 18:2392–400. 10.1681/ASN.2006080811 17599974

[13] Manivel JC Sibley RK Dehner LP. Complete and incomplete Drash syndrome: a clinicopathologic study of five cases of a dysontogenetic-neoplastic complex. Hum Pathol. (1987) 18:80–9. 10.1016/S0046-8177(87)80199-5 3028928

[14] Sherbotie JR van Heyningen V Axton R Williamson K Finn LS Kaplan BS. Hemolytic uremic syndrome associated with Denys-Drash syndrome. Pediatr Nephrol. (2000) 14:1092–7. 10.1007/s004670000389 11045393

[15] Alge JL Wenderfer SE Hicks J Bekheirnia MR Schady DA Kain JS. Hemolytic uremic syndrome as the presenting manifestation of WT1 mutation and Denys-Drash syndrome: a case report. BMC Nephrol. (2017) 18:243. 10.1186/s12882-017-0643-1 28720077 PMC5516385

[16] Yang AH Chen JY Chen BF. The dysregulated glomerular cell growth in Denys-Drash syndrome. Virchows Arch. (2004) 445:305–14. 10.1007/s00428-004-1069-2 15232745

[17] Morrison AA Viney RL Saleem MA Ladomery MR. New insights into the function of the Wilms tumor suppressor gene WT1 in podocytes. Am J Physiol Renal Physiol. (2008) 295:F12–7. 10.1152/ajprenal.00597.2007 18385267

[18] Chen G Liu H Liu F. A glimpse of the glomerular milieu: from endothelial cell to thrombotic disease in nephrotic syndrome. Microvasc Res. (2013) 89:1–6. 10.1016/j.mvr.2013.06.011 23851046

[19] Hu YF Tan Y Yu XJ Wang H Wang SX Yu F. Podocyte Involvement in Renal Thrombotic Microangiopathy: A Clinicopathological Study. Am J Nephrol. (2020) 51:752–60. 10.1159/000510141 32862175

[20] Katavetin P Katavetin P. VEGF inhibition and renal thrombotic microangiopathy. N Engl J Med. (2008) 359:205–7. 10.1056/NEJMc080770 18614790

[21] Eremina V Jefferson JA Kowalewska J Hochster H Haas M Weisstuch J. VEGF inhibition and renal thrombotic microangiopathy. N Engl J Med. (2008) 358:1129–36. 10.1056/NEJMoa0707330 18337603 PMC3030578

[22] Noris M Mele C Remuzzi G. Podocyte dysfunction in atypical haemolytic uraemic syndrome. Nat Rev Nephrol. (2015) 11:245–52. 10.1038/nrneph.2014.250 25599621

[23] Keir LS Firth R Aponik L Feitelberg D Sakimoto S Aguilar E. VEGF regulates local inhibitory complement proteins in the eye and kidney. J Clin Invest. (2017) 127:199–214. 10.1172/JCI86418 27918307 PMC5199702

[24] Valoti E Alberti M Tortajada A Garcia-Fernandez J Gastoldi S Besso L. A novel atypical hemolytic uremic syndrome-associated hybrid CFHR1/CFH gene encoding a fusion protein that antagonizes factor H-dependent complement regulation. J Am Soc Nephrol. (2015) 26:209–19. 10.1681/ASN.2013121339 24904082 PMC4279739

[25] Zipfel PF Edey M Heinen S Józsi M Richter H Misselwitz J. Deletion of complement factor H-related genes CFHR1 and CFHR3 is associated with atypical hemolytic uremic syndrome. PLoS Genet. (2007) 3:e41. 10.1371/journal.pgen.0030041 17367211 PMC1828695

