# Supplementary material for: Case Report: Denys–Drash Syndrome With WT1 Causative Variant Presenting as Atypical Hemolytic Uremic Syndrome
Source: Front Pediatr. 2020 Dec 18;8:605889. doi: 10.3389/fped.2020.605889 (PMC7775732; doi:10.3389/fped.2020.605889)
Supplement: Supplementary file 1 [file Data_Sheet_1.pdf]

Supplementary Materials

Bioinformatics analysis and variants selection

The raw data of genome sequencing was saved as FASTQ format followed by the bioinformatics analysis. First of all, Illumina adapter sequences and low-quality reads (< 80bp) were filtered by Cutadapt. Pre-processed sequences were then mapped to human reference genome hg19 (UCSC) using BWA. Duplicated reads were removed by Picard while mapping reads were prepared for variation detection. Secondly, single nucleotide polymorphisms (SNPs) and insertion–deletion mutations (indels) were detected by GATK HaplotypeCaller and detected variants would be filtered by GATK VariantFiltration according to the following parameters: --filterExpression “MQ0 >= 4 && ((MQ0 / (1.0 * DP)) > 0.1)” --filterName “HARD_TO_VALIDATE” --filterExpression “DP < 5” –filterName “LowCoverage” --filterExpression “QUAL < 30.0” --filterName “VeryLowQual” --filterExpression “QUAL > 30.0 && QUAL < 50.0” --filterName “LowQual” --filterExpression “QD< 1.5” --filterName “LowQD” --filterExpression “FS > 10.0” --filterName “StrandBias”. By finishing the above processes, data would be transformed to VCF format. Variants were further annotated by ANNOVAR, linked to multiple databases including 1000 Genomes, ESP6500, dbSNP, EXAC, Inhouse (MyGenostics), HGMD and predicted by SIFT, PolyPhen-2, MutationTaster and GERP++.

Variants selection was progressed by following these five steps to obtain the potential pathogenic variations: a) variation counts should ＞ 5, variation ration should be ⩾ 30%; b) remove those variations, the frequency of which showed more than 5% in 1000g, ESP6500 and Inhouse databases; c) remove variations in InNormal database (MyGenostics); d) Remove the synonyms except for those had been reported in HGMD. Selected variants would be then interpretated.
